# Extemporaneous Vesicant

**Published:** 1844-07

**Authors:** 


*Extemporaneous Vesicant.* By Dr. Darcq.—Into a flat watch glass, pour from 8 to 10 drops of very concentrated ammonia; cover the liquid with a large piece of linen on a rather less diameter than that of the glass, and slowly apply this little apparatus to the previously sheaved skin. Keep the whole in its place by means of moderate pressure with the fingers.

As soon as a red ring, about 2 *centimetres* in breadth, is observed round the glass, it is certain that vesication is effected. Sometimes scarcely 30 seconds are necessary for obtaining this result. It remains only to remove the apparatus, to wash the part, and to tear away with a pair of nippers the epidermis, which comes off easily and in one piece.

The dressing is according to the object in view,—to the indications of the endermic method for example.—*Chemist from Bull. de Therap.—Am. Jour. of Pharmacy.*

